# Validated Negative Regions (VNRs) in the VISTA Database might be Truncated Forms of Bona Fide Enhancers

**DOI:** 10.1002/ggn2.202300209

**Published:** 2024-05-16

**Authors:** Pengyu Ni, Siwen Wu, Zhengchang Su

**Affiliations:** ^1^ Department of Bioinformatics and Genomics the University of North Carolina at Charlotte Charlotte NC 28223 USA; ^2^ Present address: Department of Molecular Biophysics & Biochemistry Yale University New Haven CT 06520 USA

**Keywords:** cis‐regulatory modules, enhancers, evolution, humans, mice

## Abstract

The VISTA enhancer database is a valuable resource for evaluating predicted enhancers in humans and mice. In addition to thousands of validated positive regions (VPRs) in the human and mouse genomes, the database also contains similar numbers of validated negative regions (VNRs). It is previously shown that the VPRs are on average half as long as predicted overlapping enhancers that are highly conserved and hypothesize that the VPRs may be truncated forms of long bona fide enhancers. Here, it is shown that like the VPRs, the VNRs also are under strong evolutionary constraints and overlap predicted enhancers in the genomes. The VNRs are also on average half as long as predicted overlapping enhancers that are highly conserved. Moreover, the VNRs and the VPRs display similar cell/tissue‐specific modification patterns of key epigenetic marks of active enhancers. Furthermore, the VNRs and the VPRs show similar impact score spectra of in silico mutagenesis. These highly similar properties between the VPRs and the VNRs suggest that like the VPRs, the VNRs may also be truncated forms of long bona fide enhancers.

## Graphical abstract

The VISTA enhancer database contains thousands of validated positive regions (VPRs) and validated negative regions (VNRs). It is suggested that VPRs may be truncated forms of long bona fide enhancers. As VNRs exhibit similar evolutionary constraints and chromatin modification patterns to VPRs, like VPRs, VNRs may be also truncated forms of long bona fide enhancers.

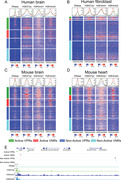

## 1 Introduction

Cis‐regulatory modules (CRMs) including enhancers, silencers, and insulators, play crucial roles in gene transcriptional regulation in development,^[^ ^1^, ^2^ ^]^ physiological homeostasis,^[^ ^3^ ^]^ diseases,^[^ ^4^ ^]^ and evolution.^[^ ^1^ ^]^ However, the current understanding of CRMs is still limited, hampering the understanding of these important biological processes. Therefore, one of the major focuses in current research in genomics is to characterize all the CRMs in the genomes of humans and other important organisms such as mice.^[^ ^5^, ^6^ ^]^ Although numerous computational methods have been developed to predict CRMs at a genome scale^[^ ^7^, ^8^, ^9^, ^10^, ^11^, ^12^, ^13^, ^14^ ^]^ (for a review see^[^ ^15^, ^16^ ^]^), predicted CRMs by different methods have little in common.^[^ ^17^ ^]^ Large‐scale experimental validation of these predicted CRMs is necessary, however, this remains a challenging task, as results from high throughput methods such as STARR‐seq have little overlap with predicted enhancers based on epigenetic marks.^[^ ^18^ ^]^ Moreover, these high throughput methods can only assess short (≈500 bp) CRMs^[^ ^19^, ^20^, ^21^ ^]^ in episomal expression vectors, resulting in high false positive and false negative rates.^[^ ^22^, ^23^ ^]^ Alternatively, the accuracy of predicted CRMs can be evaluated by using a large gold standard set of experimentally determined or validated CRMs in the genomes. The VISTA enhancer database^[^ ^24^ ^]^ is probably the most comprehensive and valuable resource for such purposes. However, experimentally validated human and mouse enhancers in the VISTA database are limited by their relatively small numbers and bias to ultra‐conserved ones.^[^ ^24^ ^]^ Moreover, correct determination of the boundaries of CRMs is crucial, but also notoriously difficult,^[^ ^17^, ^25^ ^]^ because even truncated forms of a long CRM might show varying levels of activities in transgene animal models.^[^ ^1^, ^26^, ^27^ ^]^ It is also technically difficult to assess long enhancers in transgene animal models at a large scale. This problem is further complicated by the fact that functionally related independent enhancers may cluster together to form super‐enhancers,^[^ ^28^, ^29^ ^]^ or locus control regions (LCRs),^[^ ^30^ ^]^ which can be tens of thousands of base pairs long. Consequently, although most VISTA enhancer candidates were carefully chosen for experimental validation based on their conservation levels and TF binding profiles,^[^ ^24^ ^]^ they might not necessarily be in correct lengths. An expansion of experimentally validated enhancers with correctly defined boundaries will greatly facilitate the development of computational methods to accurately predict CRMs, in particular, enhancers in the genomes.

In a recent effort to validate our predicted CRM candidates (CRMCs) in the human^[^ ^11^ ^]^ and mouse^[^ ^12^ ^]^ genomes, our CRMCs are able to recall almost all of the human and mouse “validated positive regions (VPRs)” (i.e., VISTA enhancers)^[^ ^24^ ^]^ located in the human and mouse genome regions that our algorithms are targeted to. However, the recalled human and mouse VISTA enhancers are on average half as long as the recalling CRMCs. The parts of our recalling CRMCs that the recalled VISTA enhancers lack are under strong evolutionary constraints, suggesting that many VISTA enhancers might be only parts of longer enhancers whose truncated forms are still at least partially functional under the conditions assessed.^[^ ^11^, ^12^ ^]^ At the same time, we find that many VISTA enhancers may contain a small portion of non‐enhancer sequences. Thus, the boundaries of these VPRs might need to be redefined, and our results suggest that most current VISTA enhancers might be much longer than originally thought.

Interestingly, the VISTA database also includes a similar number of so‐called “validated negative regions (VNRs)” as the VPRs (i.e., VISTA enhancers).^[^ ^24^ ^]^ These VNRs failed to show enhancer activities in transgene animals under the conditions tested. However, this does not mean that all these VNRs are not enhancers or silencers.^[^ ^24^ ^]^ In this study, we found that both human and mouse VNRs in the VISTA database share all the properties that we noted for the VISTA enhancers/VPRs. We therefore hypothesize that most of these VNRs might be functional at developmental stages or physiological conditions that have not been evaluated and that like VPRs, these VNRs also might be much longer than documented in the VISTA database.

## 2 Experimental Section

944 VNRs of humans and 504 VNRs of mice were downloaded from the VISTA database (access date 06/03/2022).^[^ ^24^ ^]^ VNRs located in the genome regions covered by available TF Chip‐seq datasets downloaded from Cistrome DB were then retained,^[^ ^31^ ^]^ which were used for predicting the CRMCs.^[^ ^11^, ^12^ ^]^ In total, 858 (90.9%) VNRs of humans and 415 (82.3%) VNRs of mice were kept for analysis. 1426947 and 912197 predicted CRMCs in the human (version 2) and mouse (version 2) genomes, respectively, were downloaded from the PCRMs database^[^ ^32^ ^]^ at https://cci‐bioinfo.uncc.edu. The phyloP scores which describe basewise conservation of the human (hg38 phyloP100way) and the mouse genomes (mm10 phyloP60way) from the UCSC genome browser were downloaded.^[^ ^33^ ^]^

### 2.1 Generation of Control Sequences for Recalling VPRs and VNRs

**Figure 1:**
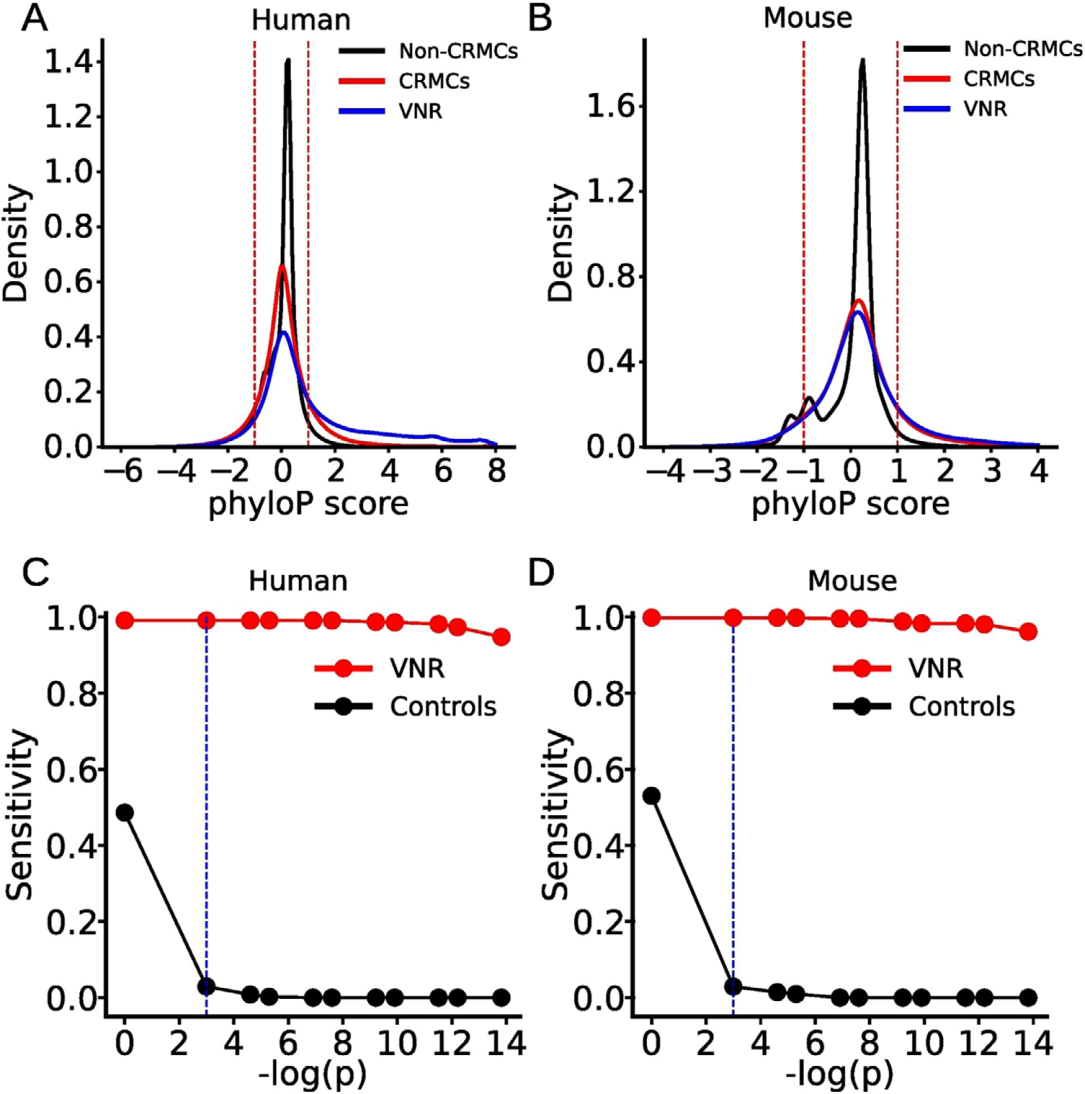
Relationship between our predicted CRMCs and the VNRs. A, B) Distributions of phyloP scores of the VNRs, the predicted CRMCs and non‐CRMCs of A) humans and B) mice. C, D) Sensitivity (recall rate) of our CRMCs or the control sequences as a function of the *p*‐value cutoff for recalling VNRs of C) humans D) and mice. The dashed vertical lines indicate the *p*‐value cutoff of 0.05. The sensitivity of the CRMCs predicted at all the indicated *p*‐value cutoffs is significantly higher (*p* < 2.2 × 10^−302^, χ^2^ test) than the control sequences for recalling the VISTA enhancers/VPRs and VNRs.

To create a set of control sequences for recalling VNRs in a genome used in **Figure** **1**C,D, for each predicted CRMC, a sequence segment with the same length as the CRMC from the genome regions covered by TF ChIP‐seq binding peaks used to predict the CRMCs was randomly selected.^[^ ^11^, ^12^ ^]^

### 2.2 Prediction of the Functional States of the VPRs and VNRs in Various Tissues

To infer the activities of the VPRs and the VNRs across different cell/tissue types in humans and mice, firstly, the functional states of the human and mouse CRMCs in 67 human and 64 mouse cell/tissue types were predicted using a machine‐learning model UFSP that uses signals of four epigenetic marks on the sequences.^[^ ^34^ ^]^ Then, the functional states of a VISTA element were predicted in each tissue based on the functional states of its overlapping CRMCs. A CRMC and a VISTA element were considered overlapping if they have at least 50% reciprocal overlap in their lengths. To see whether the VPRs and the VNRs have similar cell/tissue‐specific activation patterns, two‐way clustering of their predicted functional states in the human and mouse cell/tissue types was performed.

### 2.3 In Silico Mutagenesis Analyses of VPRs and VNRs

To identify the functional impacts of point mutations in the VPRs and the VNRs for their transcription factor binding or chromatin modifications in different tissues, in silico mutagenesis analyses for the human and mouse VPRs and VNRs using the deepSEA algorithm were performed.^[^ ^35^ ^]^ First, for each VPR and VNR, a 2000 bp genomic sequence was extracted from the midpoint of each element to include flanking context if the length of the element was shorter than 2000 bp and fit the default sequence length required by the deepSEA algorithm. The human hg38 and mouse mm10 reference genomes were downloaded from the UCSC genome browser and used for sequence extraction for the corresponding VPRs and VNRs. Finally, the extended DNA sequences were submitted to the deepSEA webserver https://hb.flatironinstitute.org/deepsea/?analysis = insilico using the Beluga model 2019 to perform in silico mutagenesis analysis for the extracted VRPs and VNRs. Finally, the absolute impact scores of point mutations were computed at each nucleotide position in a VPR or a VNR on TF binding, chromatin modifications, and chromatin accessibility of the VPR or the VNR in various cell/tissue types by mutating it to the other three nucleotides and chose the highest score of the three mutations as the final absolute impact score of the nucleotide position.

## 3 Results

### 3.1 VNRs in the VISTA Database Might Have Cis‐Regulatory Functions

To evaluate the potential regulatory functions of the human and mouse VPRs, we compare them with our recently predicted maps of CRMCs and non‐CRMCs in 85.5% of the human^[^ ^11^ ^]^ and 79.9% of the mouse^[^ ^12^ ^]^ genome regions covered by available TF ChIP‐seq binding peaks in various cell/tissue types (https://cci‐bioinfo.uncc.edu).^[^ ^32^ ^]^ In both the human (Figure 1A) and mouse (Figure 1B) genomes, the predicted non‐CRMCs are largely selectively neutral as indicated by the sharp zero‐peaked distributions of their phyloP conservation scores,^[^ ^36^ ^]^ suggesting that the non‐CRMCs are unlikely functional. In contrast, our predicted CRMCs tend to be under evolutionary selection, particularly, negative selection as indicated by the right‐skewed distributions of their phyloP scores (Figure 1A,B), suggesting that the CRMCs in both genomes are likely functional. Interestingly, similar to the cases of the VISTA enhancers/VPRs,^[^ ^11^, ^12^ ^]^ the VNRs in both human (Figure 1A) and mouse (Figure 1B) genomes show more right‐skewed distributions of phyloP scores than those of the predicted CRMCs, suggesting that the VNRs in both the human and mouse genomes are under stronger negative selection than most of the predicted CRMCs.^[^ ^34^ ^]^ These results are consistent with the fact that, like the VISTA enhancers/VPRs, the VNPs in both the human and mouse genomes are mostly ultra‐conserved elements selected for validation of enhancer activities in transgene mice.^[^ ^26^ ^]^ Our predicted human and mouse CRMCs at a *p*‐value cutoff of 5 × 10^−5^ recalled 846 (98.6%) of the 858 human VNRs (Figure 1C; Tables S1, Supporting Information) and 408 (98.3%) of the 415 mouse VNRs (Figure 1D; Tables S2, Supporting Information), located in the ChIP‐seq peak‐covered human and mouse genome regions, respectively. In contrast, the control sequences with matched lengths that are randomly selected from the peak‐covered genome regions only recall a significantly small portion (<5%) of the VNRs in both genomes (p < 2.2×10^−302^, χ^2^ test). Thus, like the VISTA enhancers/VPRs,^[^ ^11^, ^12^ ^]^ the VNRs also are large subsets of our predicted CRMCs in both the human and mouse genomes. We therefore hypothesize that the VNRs might be functional under certain developmental stages and/or physiological homeostasis yet to be tested as argued earlier.^[^ ^37^ ^]^

### 3.2 VNRs Might Be the Most Conserved Parts of Longer Bona Fide Enhancers

**Figure 2:**
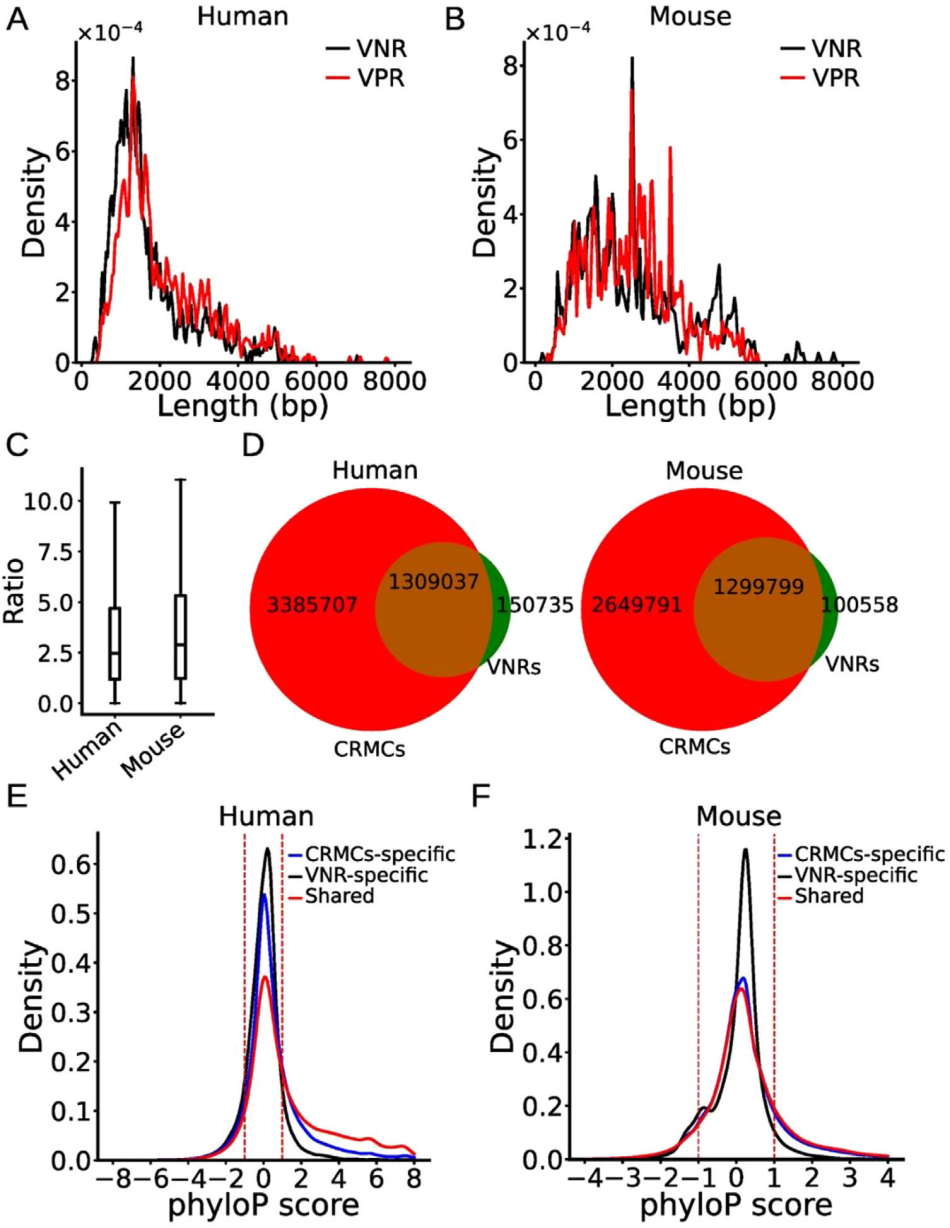
Properties of the VNRs in the human and mouse genomes. A, B) Distributions of the lengths of the VPRs and the VNRs in the A) human and B) mouse genomes. C) Boxplots of the ratio of the length of a recalled CRMC over that of its recalled VNRs of humans and mice. D) Venn diagram showing the number of nucleotide positions shared by recalling CRMCs and recalled VNRs and the number of positions specific to the recalling CRMCs and the recalled VNRs in the human and mouse genomes. E, F). Distributions of phyloP scores of the positions shared by the recalling CRMCs and the recalled VNRs, and of the positions specific to the recalling CRMCs and the recalled VNRs of E) humans and F) mice. The differences between the distributions of phyloP scores of the shared and VNR‐specific positions in both the human and mouse genomes are significantly different, *p* < 2.2 × 10^−302^, K‐S test.

The VNRs and the VISTA enhancers/VPRs have similar length distributions with a mean length of 1741 and 2079 bp for humans (**Figure** **2**A) and 2612 and 2520 bp for mice (Figure 2B), respectively. As in the cases of the VISTA enhancers/VPRs,^[^ ^11^, ^12^ ^]^ the recalled VNRs in both genomes also are on average half as long as the recalling CRMCs (Figure 2C). Moreover, the vast majority of human (89.7%) and mouse (89.2%) VNR positions overlap those of their recalling CRMCs. Meanwhile, 10.3% and 10.8% of the human and mouse VNR positions, respectively, are missed by their recalling CRMCs (Figure 2D). To see whether we over‐predicted the lengths of the VNRs, or the VNRs were only parts of longer enhancers, we compared phyloP conservation scores of positions shared by recalling CRMCs and recalled VNRs, specific to the recalling CRMCs, and specific to the recalled VNRs. In both human (Figure 2E) and mouse (Figure 2F) genomes, positions shared by the CRMCs and the VNRs as well as CRMC‐specific positions (72.1% for humans and 76.1% for mice) tend to be under evolutionary constraints, suggesting that the positions in recalling CRMCs that the recalled VNRs lack might be functional. Thus, the VNRs might be the only components of longer enhancers. It is interesting to test whether the lack of required parts is the reason that the VNRs did not show enhancer activities under the conditions tested. In contrast, in both human (Figure 2E) and mouse (Figure 2F) genomes, the VNRs specific positions (10.3% for humans and 10.8 for mice) tend to be selectively neutral, suggesting that the positions in the recalled VNRs that the recalling CRMCs lack might not be functional. Therefore, although the recalled VNRs are on average only half as long as the recalling CRMCs, they might contain a small portion of non‐enhancer sequences. Based on these results, we hypothesize that the VNRs might be the most conserved parts of longer bona fide enhancers.

### 3.3 Both VPRs and VNRs Show Tissue‐Specific Activities in Various Cell/Tissue Types

**Figure 3:**
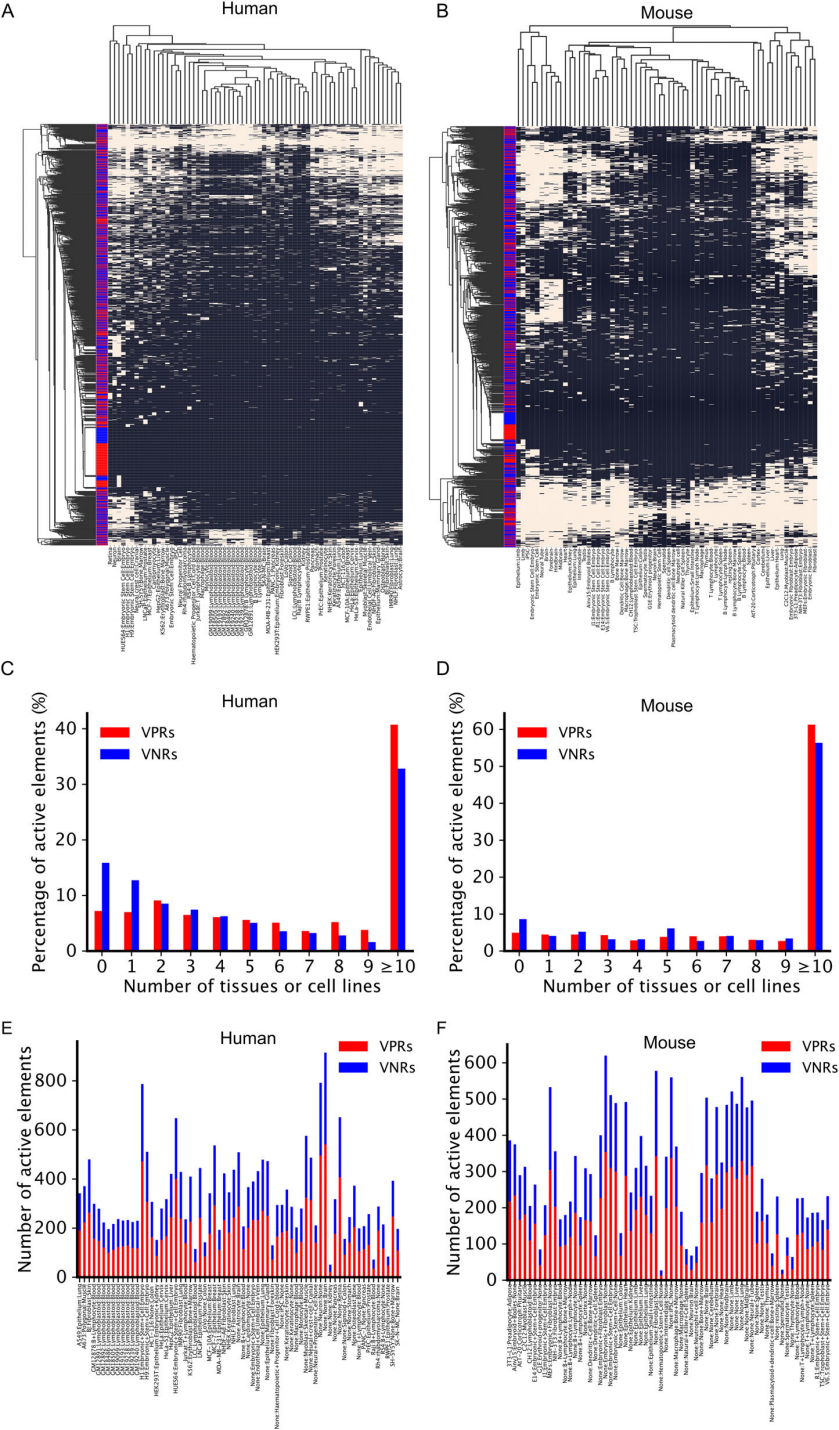
Tissue‐specific activities of the VPRs and VNRs across multiple cell/tissue types. A, B) Heatmaps of two‐way hierarchical clustering results of the activation probabilities of the A) human and B)mouse VPRs and VNRs across multiple cell/tissue types. The red and blue bars on the left of the heatmap represent VPRs and VNRs, respectively. C, D) Percentages of the C) human and D) mouse VPRs and VNRs that are active in the indicated numbers of cell/tissue types. The white color shows the active elements, while the black shows the non‐active elements. E, F) Numbers of active VPRs and VNRs in each cell/tissue type of E) human and F) mouse.

We reason that although the human and mouse VPRs and VNRs have been mainly validated or failed to be validated, respectively, in earlier stages of mouse embryos, they might be activated in some cell lines and terminal tissues as enhancers are often reused in different contexts.^[^ ^1^, ^38^ ^]^ We also observed that both the human (**Figure** **3**A) and mouse (Figure 3B) cell/tissue types are clustered together according to their lineage relationships, indicating that the functional states of the VPRs and the VNRs reflect the identity of the cell/tissue types as we have demonstrated earlier.^[^ ^34^ ^]^ Moreover, both the human (Figure 3A) and mouse (Figure 3B) VRPs and VNRs are often clustered together based on their functional states in the corresponding cell/tissue types. These results indicate that both the human and mouse VRPs and VNRs indeed have highly similar cell/tissue‐specific activation patterns. Furthermore, varying proportions of both the human (Figure 3C) and mouse (Figure 3D) VPRs and VNRs are active in different numbers of corresponding cell/tissue types, and more than 92.8% and 95% of human and mouse VPRs, respectively, and more than 84.1% and 91.4% of human and mouse VNRs, respectively, are active in at least one of the corresponding cell/tissue types. Also, varying numbers of both the human (Figure 3E) and mouse (Figure 3F) VPRs and VNRs are active in all the corresponding tissues/cell lines. In summary, like most of the human and mouse VPRs, most of the human and mouse VNRs display cell/tissue‐specific activation and thus might be functional.

### 3.4 Chromatin Modifications in Both VPRs and VNRs Reflect Their Functional States in Cells/Tissues

**Figure 4:**
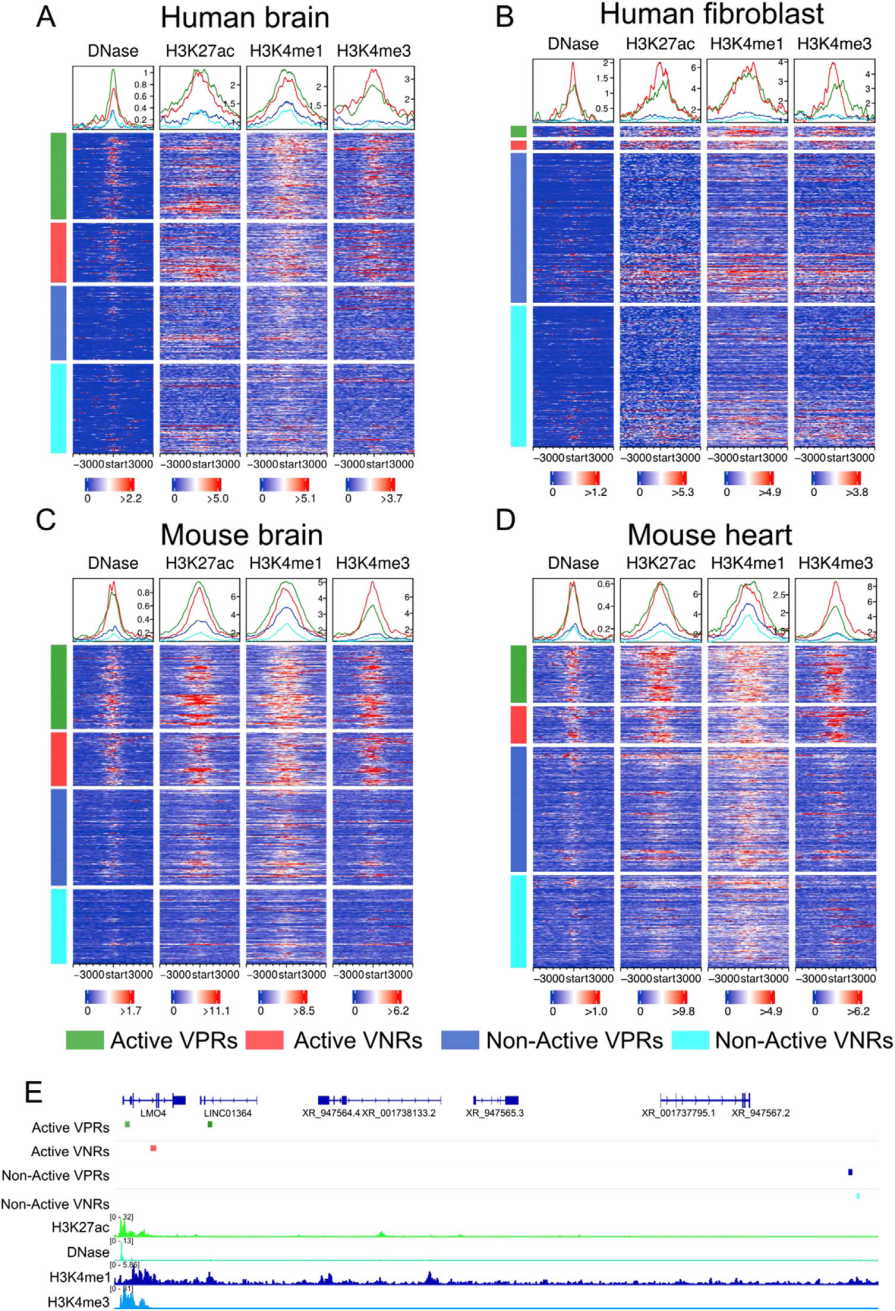
Examples of epigenetic signals in activated and non‐activated human and mouse VPRs and VNRs in four cell/tissue types. A–D) Heatmaps of the DNase and three histone marks signals in activated and non‐activated VPRs and VNRs in the A) human brain tissue and B) fibroblast cells, and in the C) mouse brain tissue and D) heart tissue. E) Signal tracks of the four epigenetic marks along a region in human chromosome 1 in the brain tissue, containing two active VPRs, an active VNR, a non‐active VPR, and a non‐active VNR.

We found that the predicted functional states of both the human and mouse VPRs and VNRs are reflected by their epigenetic marks in the corresponding cell/tissue types. Shown in **Figure** **4**A–D are examples of two tissues in humans (brain and fibroblast) and in mice (brain and heart). Specifically, both active VPRs and active VNRs in humans (Figure 4A,B) and mice (Figure 4C,D) showed stronger signals of chromatin accessibility as measured by DNase‐seq and key histone marks of active enhancers (H3K27ac, H3K4me1, and H3K4me3). In contrast, the non‐active VPRs and non‐active VNRs in humans (Figure 4A,B) and mice (Figure 4C,D) have weak signals of chromatin accessibility and the key histone marks of active enhancers. Shown in Figure 4E are examples of signal tracks of the four epigenetic marks along a region on chromosome 1 in the human brain tissue with two active VPRs, an active VNP, a non‐active VPR, and a non‐active VNR. Taken together, these results indicate that both VPRs and VNRs in humans and mice possess similar chromatin modification patterns for active enhancers in corresponding specific cell/tissue types, and thus, might be functional in them.

### 3.5 Mutations in VNRs and VPRs Show Similar Functional Impacts

**Figure 5:**
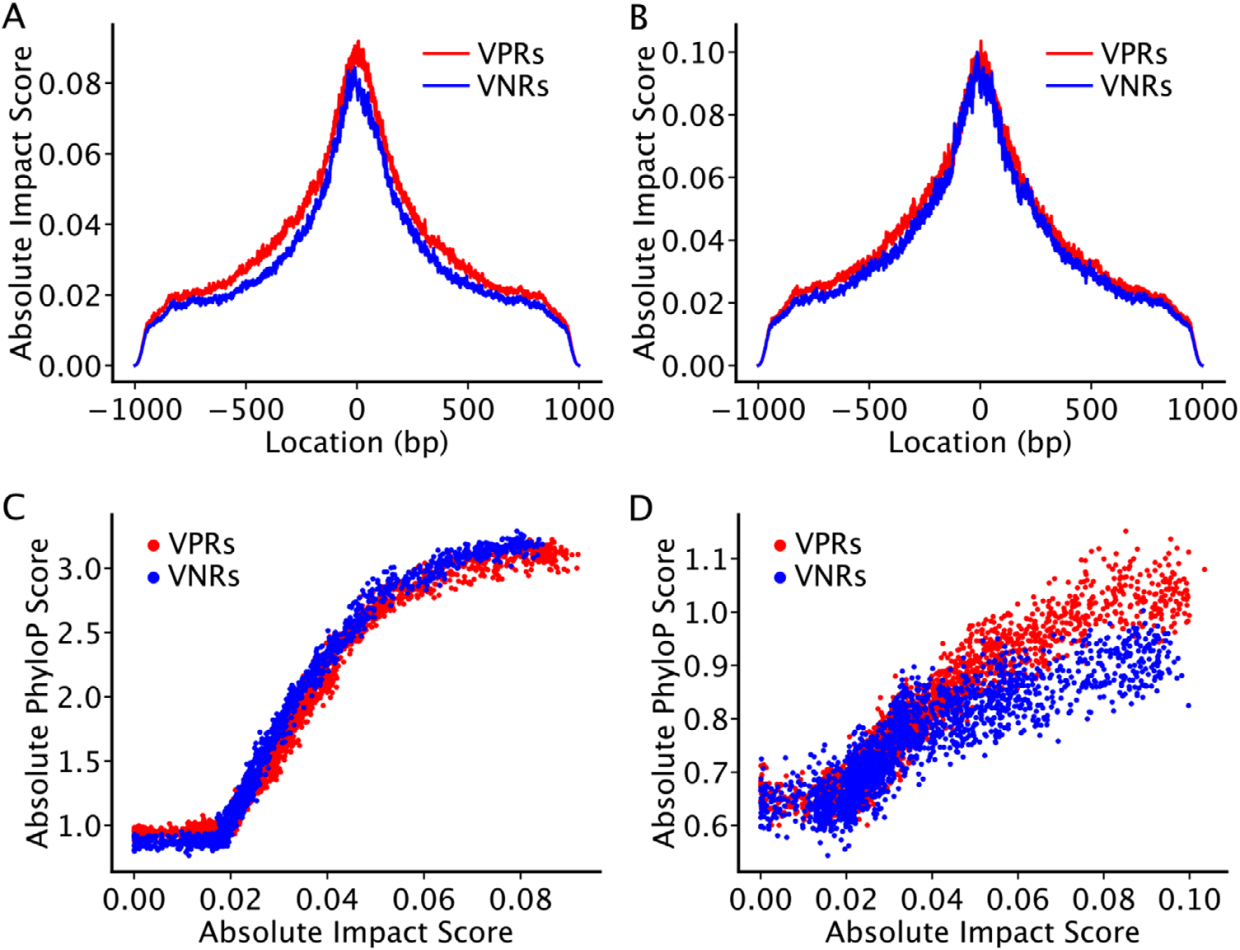
In silico mutagenesis analyses of the human and mouse VNRs and VPRs. A, B) Average absolute impact scores at each nucleotide position across all the A) human and B) mouse VNRs or VPRs. C, D) Correlation between the average absolute phyloP scores and average absolute impact scores along C) human or D) mouse VNRs and VPRs.

To compare the importance of nucleotides in the human and mouse VPRs and VNRs, we conducted in silico mutagenesis analyses for each nucleotide position in each of the VPRs and VNPs using the Beluga model 2019^[^ ^35^ ^]^ (Experimental Section). As shown in **Figure** **5**A,B, the average absolute impact scores at each position across all the VPRs and VNRs peak at the midpoints and gradually decrease outward to the 500 bp flanking regions on either side, suggesting that the central parts of both VPRs and VNRs are more critical than the flanking parts. The consistency between the distributions of absolute impact scores of the VPRs and VNRs suggests that the nucleotides of the VNRs are as critical as those in the VPRs for TF binding, chromatin modifications, and chromatin accessibility. Moreover, we analyzed the relationship between the average absolute impact scores and average conservation scores at each position across all the VPRs and VNPs. To this end, we took the absolute values of the phyloP conservation scores since both high negative values (indicating accelerated evolution) and high positive values (indicating conservation) can imply functionality. As shown in Figure 5B,D, the average absolute phyloP scores are highly correlated with the absolute impact scores of VPRs and VNRs after the removal of evolutionarily neutral positions with a low average absolute phyloP score (<1.0 for humans and < 0.7 for mice). Taken together, the similar mutation impact scores of the VPRs and VNRs again suggest that like the VPRs, the VNRs might also be functional.

## 4 Discussion

The experimentally validated human and mouse enhancers in the VISTA database are a valuable resource for evaluating predicted CRMCs in the human and mouse genomes. However, based on evolutionary evidence, we hypothesize that the VISTA enhancers/VPRs of both humans and mice might be only parts of otherwise longer enhancers,^[^ ^11^, ^12^ ^]^ which might be functional in their truncated forms under developmental stages and/or physiological conditions tested. Moreover, both the human and mouse VNRs in the VISTA database share all the properties that we noted for the human and mouse VISTA enhancers/VPRs.^[^ ^11^, ^12^ ^]^ First, almost all the VPRs and the VNRs in the VISTA database are recalled by our predicted CRMCs that are largely under evolutionary constraints. Second, both recalled VPRs and VNRs are on average half as long as the recalling CRMCs. Third, the parts in the recalling CRMCs that the recalled VPRs or VNRs lack tend to be under purifying selection. Fourth, both the VPRs and the VNRs might include a small portion of non‐enhancer sequences. Fifth, both the VPRs and the VNRs are under more evolutionary constraints than average CRMCs. Sixth, both the VPRs and the VNRs display cell/tissue‐specific activities. Seventh, functional states of both the VPRs and the VNRs are reflected by their similar epigenetic modification patterns. Finally, both the VPRs and the VNRs show similar impact spectra of mutagenesis, suggesting that they have similar sensitivities to mutations and corrective mechanisms. Further studies are required to uncover specific links between these score profiles and their impact on enhancer functions. Based on these similarities, we hypothesize that like the VPRs, the VNRs might also be the most conserved parts of longer bona fide CRMCs, which might be functional in their full lengths or under developmental stages and/or physiological conditions that remain to be tested. To test these hypotheses, we need to redefine the boundaries of both the VPRs and the VNRs, which most likely results in longer enhancers. At the same time, we need to validate the appropriately extended VNRs under more developmental stages and/or physiological conditions that have not been examined before. Through our previous studies^[^ ^11^, ^12^ ^]^ and the current study, we provide candidate sets (Tables S1 and S2, Supporting Information) for such efforts. An expanded gold standard set of enhancers with correctly defined boundaries will greatly facilitate the development of computational methods to predict all CRMCs in the genomes.

## Conflict of Interest

The authors declare no conflict of interest.

## Author Contributions

P.N. and Z.S. conceived the project and wrote the manuscript. P.N. and S.W. carried out all computational experiments and analyses. All authors read and approved the final manuscript.

## Supporting information

Supporting Information

## Data Availability

The data that support the findings of this study are available from the corresponding author upon reasonable request.
